# A Qualitative Analysis of Opportunities to Strengthen Coordination Between Humanitarian Mine Action and Emergency Care for Civilian Casualties of Explosive Injury

**DOI:** 10.1017/dmp.2025.30

**Published:** 2025-02-20

**Authors:** Hannah B. Wild, Micah Trautwein, Constance Fontanet, Elke Hottentot, Sebastian Kasack, Alex Munyambabazi, Emilie Calvello-Hynes, Adam Kushner, Barclay Stewart

**Affiliations:** 1 Department of Surgery, University of Washington, Seattle, WA, USA; 2 Explosive Weapons Trauma Care Collective, International Blast Injury Research Network, University of Southampton, Southampton, UK; 3 Dartmouth Geisel School of Medicine, Hanover, NH, USA; 4 Victim Assistance Specialist, Geneva, Switzerland; 5 Mines Advisory Group, Manchester, UK; 6 Amputee Self Help Network Uganda, Kampala, Uganda; 7 World Health Organization, Geneva, Switzerland; 8 Surgeons Overseas, New York, NY; 9 Global Injury Control Section, Harborview Injury Prevention and Research Center, Seattle, WA, USA

**Keywords:** civilian casualties, conflict, explosive weapons, explosive ordnance, humanitarian mine action, blast injury, trauma care, low-resource settings

## Abstract

**Objectives:**

Explosive ordnance (EO) and explosive weapons (EW) inflict significant suffering on civilian populations in conflict and post-conflict settings. At present, there is limited coordination between humanitarian mine action (HMA) and emergency care for civilian victims of EO/EW. Key informant interviews with sector experts were conducted to evaluate strategies for enhanced engagement between HMA and emergency care capacity-building in EO/EW-affected settings.

**Methods:**

A cross-sectional qualitative study was conducted to interview HMA and health sector experts. Data were analyzed in Dedoose using deductive and inductive coding methods.

**Results:**

Nineteen key informants were interviewed representing sector experts in HMA, health, and policy domains intersecting with the care of EO/EW casualties. Recommendations included integration of layperson first responder trainings with EO risk education, development of prehospital casualty notification systems with standardized health facility capacity mapping, and refresher trainings for HMA medics at local health facilities.

**Conclusions:**

Medical capabilities within the HMA sector hold potential to strengthen emergency care for civilian EO/EW casualties yet in the absence of structured coordination strategies is underutilized for this purpose. Increased HMA engagement in emergency care may enhance implementation of evidence-based emergency care interventions to decrease preventable death and disability among civilian victims of EO/EW in low-resource settings.

Explosive ordnance (EO) and explosive weapons (EW) inflict significant suffering on civilian populations in contemporary conflicts such as those in Yemen, Gaza, Sudan, and Ukraine, in addition to the ongoing consequences of contamination in post-conflict settings.^1–3 4^ Civilian casualties of EO have a nearly 40% mortality rate.^5,6^ This figure is 5–20 times higher than that reported among blast-injured cohorts treated at military facilities or high-resource civilian centers.^7–9^ This mortality disparity is in large part due to the lack of a formalized casualty care continuum in many low-resource conflict and post-conflict settings where EO incidents occur in contrast with the well-developed trauma systems of military or high-resource civilian contexts. A range of factors including Lack of systematic community first aid response programs, limited or uncoordinated prehospital systems, underdeveloped trauma resuscitation/triage processes at facilities receiving casualties and insufficient trained surgical and anesthesia workforce all contribute to this gap. As the impact of EO including improvised explosive devices and the use of explosive weapons in populated areas affects civilians in conflicts globally, there is an urgent need for coordinated efforts to strengthen emergency care for civilian EO/EW casualties in low-resource settings.

At the 26^th^ International Meeting of Mine Action National Directors and United Nations Advisers in 2023, the Explosive Weapons Trauma Care Collective (EXTRACCT) was established for this purpose.^10^ Humanitarian mine action (HMA) falls under the Global Protection Cluster within the humanitarian cluster system and is tasked with mitigating harm from EO through a range of activities including land clearance, explosive ordnance risk education (EORE), and victim assistance. Significant medical capacity is present on demining teams through medics trained in accordance with the International Mine Action Standards (IMAS) 10.40 on Medical Support to Demining Operations and in 2021, IMAS 13.10 on Victim Assistance in Mine Action was developed.^11^ Yet at present, few structured pathways exist for engagement between HMA and health stakeholders in EO/EW-affected settings to strengthen emergency care systems and improve trauma care for the injured.^12^

A systematic review was previously conducted to identify evidence-based opportunities for enhanced coordination between HMA and health stakeholders for implementation of trauma care interventions in low-resource settings affected by EO and EW.^13^ The findings of this review were synthesized into the Civilian Casualty Care Chain (C-CCC), a framework that built upon the continuum of care for the injured delineated by the World Health Organization (WHO)’s Emergency Care System Framework and highlights opportunities for HMA stakeholder involvement in trauma care capacity building for civilian EO/EW victims. To further elucidate the findings of this review, a qualitative analysis of key informant interviews was conducted with HMA sector stakeholders. The objective of these interviews was to qualitatively evaluate the experiences, perceptions, and recommendations of sector experts to increase HMA engagement with the emergency health response to civilian casualties of EO/EW. The findings of this study may help inform the design of interventions implemented jointly by HMA and health stakeholders to strengthen emergency care and reduce preventable death and disability among EO/EW casualties in low-resource settings.

## Methods

### Study Design

A cross-sectional qualitative study was conducted using a semistructured guide to interview HMA sector experts (Supplement 1). Because victim assistance in mine action has historically focused heavily on long-term physical and psychosocial rehabilitation, this study focused on emergency care in the immediate post-injury setting. “Explosive ordnance” was defined in accordance with the IMAS 04.10 Glossary on mine action terms and “explosive weapons” was used to encompass all other forms of air- and ground-launched explosive munitions.^3,14^ An initial convenience sample of participants was identified through discussions with key HMA stakeholders including the United Nations Mine Action Service and Antipersonnel Mine Ban Convention Implementation Support Unit. Subsequent participant recruitment relied on snowball sampling. Study participants were contacted via email for recruitment and verbal informed consent was obtained. Interviews occurred between November 2021-February 2022. Interviews were conducted until data saturation was achieved (i.e., lack of new themes or information yielded).^15^

### Data Management and Ethical Protections

Participants were interviewed anonymously. Organizational affiliation and professional role were documented and maintained in a separate de-identified spreadsheet accessed only by the study lead. Audio-recorded interviews were conducted by the lead author (HW). Interviews were held individually on a secure VPN and transcribed with personal identifiers redacted. Ethical approval for this study was granted by the University of Washington Human Subjects Division.

### Data Analysis

**Figure 1.**
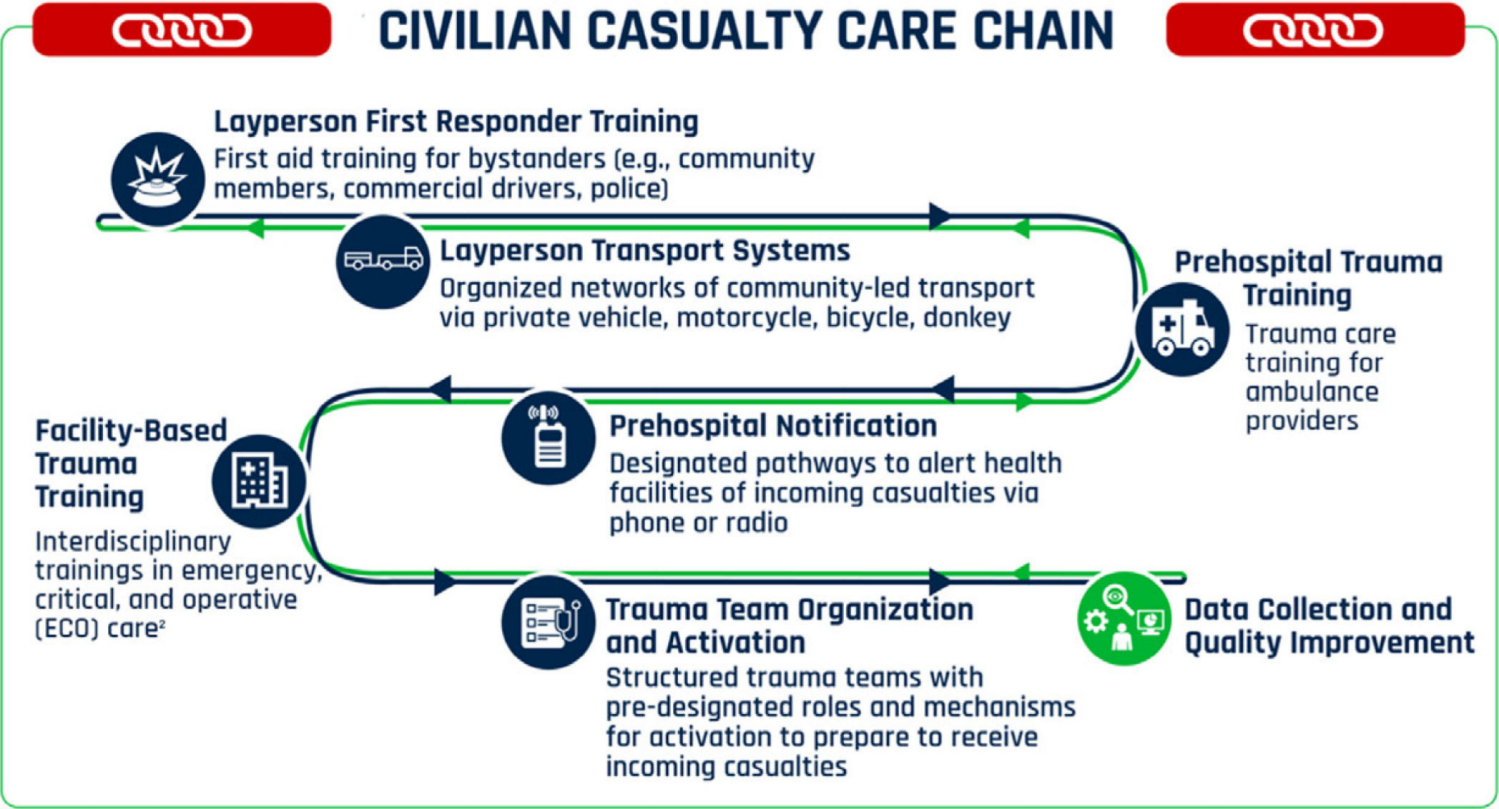
Civilian Casualty Care Chain (C-CCC)^1^ 1. The C-CCC outlines a selected set of interventions that represent opportunities for HMA stakeholders to engage in health sector initiatives to improve emergency care of EW casualties. The C-CCC is not itself an emergency care pathway, as it lacks many of the emergency care system components needed for a continuum of response. Rather, it highlights specific areas of targeted action in which HMA stakeholders might leverage their existing capabilities, infrastructure, and operations to support local emergency care systems to improve trauma care for EW casualties. 2. Interdisciplinary refers to the interprofessional nature of robust emergency, critical, and operative care, engaging all relevant health care providers including physicians, surgeons, nurses, and health officers.

**Figure 2.**
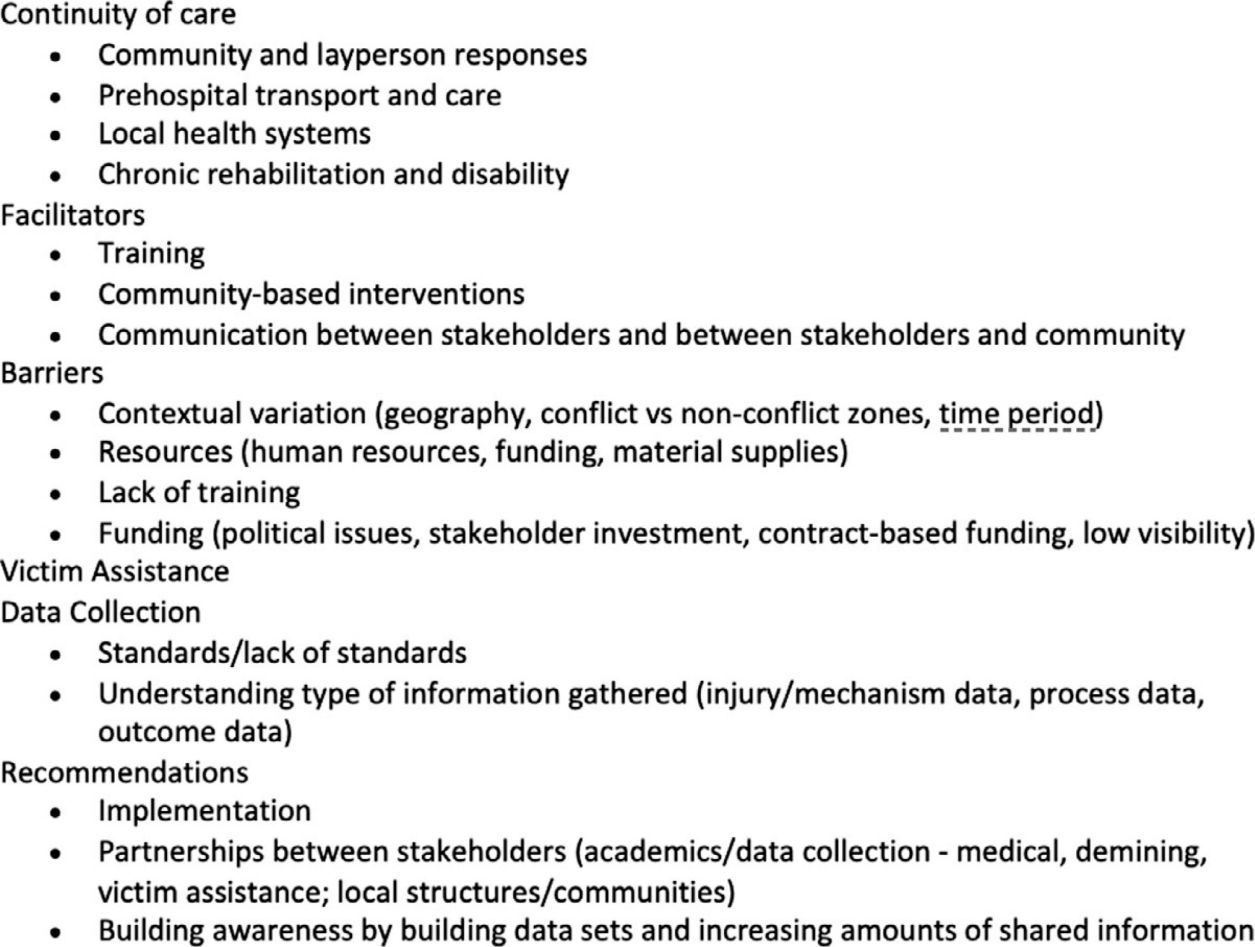
Coding tree.

Transcripts were manually evaluated for completeness and quality, and then analyzed using Dedoose 9.0.62 using deductive and inductive coding methods. Initial codes were derived directly from the interview tool (Figure 1), whereas inductive codes emerged in vivo.^16^ Each transcript was read and coded by 2 independent researchers (MT, CF). Analysis and coding occurred simultaneously until saturation was reached via an iterative process. Once generated, themes were then arranged by phase of care from point of injury to definitive care at health facility to maintain consistency with the C-CCC (Figure 2): layperson first response (i.e., bystander care prior to contact with first trained health care personnel), prehospital care (i.e., care rendered in the prehospital setting by trained health care personnel), facility-based casualty care (i.e., trauma care in the emergency unit; damage control resuscitation and surgery provided in health facilities), and system-wide measures of data collection and quality improvement.^13^

## Results

### Participant Characteristics

Nineteen key informant interviews were conducted with sector experts in HMA, emergency care, humanitarian funding agencies, policy/advocacy, and academia (Table 1). Over half of the participants were affiliated with non-governmental organizations in the HMA sector (*n*=11; 58%). Numerous themes emerged encompassing a spectrum of care from point of injury to facility-based care (Table 2).

### Layperson First Response

At the layperson first response phase, main themes included lack of a structured approach to improving bystander’s capacity to provide emergency first aid, prolonged time to reaching trained healthcare personnel, and underexplored opportunities to leverage existing local health care promotion efforts. Many EO/EW-affected regions are remote with limited organized prehospital care.^17^ Layperson first response refers to strengthening the capacity of community members and bystanders to equip them respond to life-threatening injuries (Figure 1). Participants in this study reported that currently, the needs of victims of EO- and EW-related injuries in conflict and post-conflict settings were frequently met first by local bystanders. Numerous participants recommended that training individuals in communities affected by explosive violence to perform safe extrication and basic life-saving interventions is an essential approach in regions where formal prehospital systems are resource-constrained.

“He spent 27 hours on route to get to a hospital after his injury, and that’s not an uncommon thing.”

“I think [the community] is where the first response really has to take place -- before you reach the facility and not to focus on the facility.”

Participants identified challenges to sustainability and scaling of layperson first responder (LFR) and other community-based trainings. Such challenges included a lack of clear pathways for longitudinal training as opposed to one-off interventions, access to basic equipment (e.g., personal protective equipment, hemorrhage control supplies), and contextual variation based on the local setting, conflict type, and EO/EW used. A clear opportunity for building on existing community-level interventions was identified leveraging EO risk education (EORE), a pillar of mine action through which existing community liaisons and community-based networks are established.^18^ Participants felt that existing teams of community liaisons would be well poised to deliver and sustain LFR trainings when integrated with EORE programs.

Numerous participants also highlighted the need to provide appropriate scene safety education and precautions to ensure that lay first responders did not expose themselves to risk of harm from EO/EW, unsafe infrastructure, and blood-borne pathogens. To address concerns about sustainability and integration with existing systems, several participants offered solutions including leveraging existing local personnel with relevant experience (e.g., individuals who worked as firefighters, nurses, or health care personnel prior to active conflict) or semi-formal connection with organized health systems (e.g., community health workers, civil servants) to whom educational and material support could be delivered through a train-the-trainer (ToT) approach. In addition, inconsistent bystander protection laws were viewed as a barrier to enhancing LFR capacity.

To reduce the time from point of injury to reaching a trained provider, participants raised the potential to leverage these community networks to create organized LFR transport systems using means of transportation available in the local context.^19^ At a policy level, such steps were felt to be consistent with IMAS 13.10^a^ provisions regarding collaboration with health authorities to ensure access to emergency medical transport.

### Prehospital Care

At the prehospital care phase, main themes included prolonged prehospital time prior to reaching facility-based care, lack of streamlined processes for understanding existing health system capacity, and lack of governance or coordination regarding HMA engagement with prehospital systems. In contexts where EO/EW-related injuries occur, participants frequently commented on the reality that prehospital transport can take many hours. To respond to this reality, participants felt that higher levels of training for those providing care in the prehospital setting are warranted.

Participants emphasized that establishing a clear understanding of existing medical facilities, capabilities, and capacity, including physical and human resources as well as fastest transport routes, are key for successful prehospital care (e.g., regular community-level updates/flyers about open facilities and safe passable roadways). In certain remote settings, health facilities were reported to not always be fully staffed; therefore it was recommended that such information be used to develop notification systems whereby facilities were alerted of incoming casualties to increase preparedness.

Individuals on HMA demining teams are all trained to the level of Basic Care Provider, with at least 1 Intermediate Care provider (paramedic-level trained individual) present at all times.^11^ Yet participants highlighted that, at present, there is little-to-no overlap between HMA medics and the care of civilian casualties. While demining teams feel a sense of responsibility to engage in prehospital care and transport of local accidents, injuries, or illnesses when they are coincidentally the first-on-site, there are no structured pathways for coordination between demining team medics and local emergency care providers, with the exception of nongovernmental organizations that engage in both land clearance and health provision activities (e.g., Humanity and Inclusion). Direct patient care was viewed as challenging both from a mandate and liability standpoint, which raised concerns over its acceptability and uptake:

“But that is obviously difficult…although they can work within the auspices of the [organization], if something goes wrong, that they were Samaritaning [sic] outside of that, there may be some very difficult laws about why you’re doing that when only doctors can put a line in and give tranexamic acid, we don’t even give tranexamic acid in our hospital. So what are you doing outside? So there’s, you know, there are some issues around legislation around that which make it difficult, I think what would be what would be useful to do instead is training.”

Numerous participants proposed that the integration of HMA personnel and medics into prehospital training could transform ad hoc engagement into systematic involvement in emergency care and trauma system capacity-building. For example:

“It does feel like a bit of a waste that quite well-trained medical people sat there for 8 hours a day. Clearly there’s a requirement. You can’t distract them with intensive training, but basic sort of ABCs of first aid and things like this…They could quite easily cover that without losing focus on that day-to-day task.”

Participants stated that it would be feasible to utilize medics during down-time at a safe periphery from the worksite or after-hours to conduct trainings for both lay persons and local prehospital care providers.

### Facility-Based Care

At the facility-based care phase, main themes included inadequate clinical training in the management of severely injured patients, incomplete understanding of existing local health care capacity and referral pathways, lack of essential medication and equipment and facilities in regions affected by EO/EW with implications for casualty referral, and lack of formalized communication and handover procedures. Participants raised numerous issues surrounding care at the health facility level. In the experience of participants, many of the receiving facilities for injuries of EO/EW were felt to have insufficient training and material resources to provide high-quality damage control resuscitation and damage control surgery. Participants felt that these constraints could in part be mitigated by referral pathways, communication systems, and standardized health facility capacity mapping to create shared understanding of capabilities by site. For example, several participants described successful instances in which an initial receiving hospital lacked the imaging resources or surgical skillset for definitive care of specific injuries (e.g., fractures), yet was able to expedite care through immediate resuscitation with prompt referral to a tertiary hospital for subspecialty care. Opportunities for improvement included clarification of interfacility transfer and referral pathways or shared health actor mapping:

“We need to know if there’s a local clinic… If not, well, then we need to plan our evacuation routes another way. And then we start to understand the victims’ situation.”

Currently, such connections rely on relationships between providers more than formal communication pathways:

“In terms of integration with the Ministry of Health, no -- it would be normally individual hospitals, and the way it almost inevitably runs is someone will then have someone’s mobile phone number, and we’ll call them directly. We’ll usually get an anesthetist or a surgeon’s number or the lead for the hospital’s number, because there’s that sort of individual connection that’s been made. So that’s usually how it would run…it doesn’t normally go through bureaucracy of the Ministry of Health.”

While such connections were described to have facilitated access to care in individual cases, it was not felt to be a sustainable system and, further, it lacked integration with local Ministries of Health and health systems. This coordination gap was felt to create confusion around the appropriate routing of patients, ultimately creating delays in care.

In addition to improved notification systems and capacity mapping, opportunities for facility-based training were proposed. Several participants from the HMA sector proposed that creating partnerships for bidirectional refresher trainings for the care of blast-injured patients at health facilities could be mutually beneficial to HMA medical personnel through exposure to a hospital environment to maintain their clinical skills, while also improving the trauma and burn care training of local providers:

“Better trauma care for our own mine action personnel could mean better trauma care to communities where they’re working.”

“This is pushing at an open door at the moment, because senior people in [organization] are really keen on moving this aspect of care forward… in terms of teaching and training.”

### Data Collection and Quality Improvement

Data collection was overall felt to be strong with respect to EO casualties and victims’ needs through sector-wide activities including community-based nontechnical surveys, as well as the minimum data requirements established by IMAS 5.10 on Information Management.^20^ In contrast, participants reported that these data were infrequently used to facilitate meaningful change for survivors and were not commonly operationalized into responsive programmatic changes or targeted interventions. Participants raised ethical concerns about data collection in the absence of clear pathways to improvement in services for the populations and communities from which data are being collected. As 1 participant stated,

“This humanitarian sector has been plagued…not necessarily an actual lack of data, but rather the lack of use of the data because there is a lot of gathering of data but with no clear purpose. So then there will be fatigue, of course, by both the people filling it in and also the people being asked.”

Other participants within HMA reported limitations in data analysis capacity that preclude data being fed back into evidence-based programming.

## Discussion

A qualitative analysis of key informant interviews with HMA sector stakeholders was conducted to explore opportunities and challenges related to enhanced coordination with health stakeholders to improve emergency care for civilian casualties of EO/EW. Participant responses converged on several dominant themes, each yielding concrete intervention opportunities (Table 3). Key themes and recommendations included: (i) integrated LFR and EORE trainings to enhance community-level capabilities to effectively respond to EO incidents; (ii) leveraging HMA medics to strengthen of prehospital provider skills; and (iii) centralized processes for health facility mapping linked with structured prehospital casualty notification systems and referral pathways. While HMA stakeholders can potentiate the impact of emergency care-strengthening interventions, it is ultimately local, national, and multilateral health authorities who will drive these efforts and ensure their sustainability. It is therefore imperative that collaboration with Ministries of Health, local health care facilities, and emergency, critical, and operative care providers be strengthened. At present, most of the resource allocation in victim assistance is devoted to long-term rehabilitation. Sufficient support for strengthening emergency care elements of victim assistance is required to address these gaps in coordination.

Participants reiterated the importance of bystander care given the frequency with which community members are the first to respond to EO/EW accidents in regions that are remote or with weakened health infrastructure. LFR trainings have been successfully conducted in the past among communities affected by landmines. Specifically, numerous participants cited the efforts of the Tromsø Mine Victim Center as a successful intervention during which laypersons and prehospital personnel received intensive trauma care trainings in landmine-contaminated regions of Iraq and Cambodia in the 1990s. A 5-year prospective cohort study observed a reduction in trauma-related mortality in the intervention area from approximately 40%−15%. Yet, this intervention was not scaled throughout EO/EW-affected regions globally given a lack of sector-wide integration and buy-in.^17^ An additional factor of complexity was present in the Tromsø trainings, which included a paramedic-level component for prehospital personnel including advanced skills such as endotracheal intubation. Nonetheless, by more closely integrating LFR trainings into the activities of HMA stakeholders as well as local health care providers using a ToT model, this intervention has the potential to improve implementation outcomes (e.g., uptake, penetration, sustainability).^21^ Conversely, EORE trainings could be provided to community health care workers and other health care personnel to strengthen injury prevention in EO/EW-affected settings.

Participants recommended integration with EORE activities as a key opportunity for HMA engagement in dissemination of LFR trainings. EORE is a pillar of mine action with an extensive community-facing presence through nontechnical surveys and community liaisons. To ensure a continuum of care for the injured, it is essential to ensure that trained LFRs have a clear link with the health system with established mechanisms to alert and handoff to prehospital personnel or the nearest facility. With respect to health sector engagement, LFR trainings interface directly with the objectives stipulated by the recently endorsed WHO resolution on Integrated Emergency, Critical, and Operative Care (ECO resolution.76.2), including training of community first aid responders through the WHO community first aid responder (CFAR) program.^22^

Participants in this study reported that demining team medics and paramedics possessed currently underutilized potential to contribute to trauma care trainings for local prehospital personnel. Although the primary responsibility of HMA medical personnel universally is readiness for an incident during clearance activities, on-site incidents are thankfully relatively rare. Participants envisioned numerous arrangements via which medics could be leveraged to engage in trainings for local prehospital providers without compromising their primary responsibility to ensure the safety of demining teams. Such opportunities included trainings conducted at a safe periphery from a worksite, trainings conducted after-hours, and hiring arrangements for additional medic/paramedic shifts that could be devoted to weekly trainings. HMA teams should leverage globally accepted curricula for trauma care such as the WHO/International Committee of the Red Cross Basic Emergency Care Course and specific modules on conflict-related injury.

Though such activities could theoretically require additional resource allocation, participants identified this as having mutual benefit to mine action operators through (a) the inherent desire of local HMA personnel to give back to their communities, (b) increased acceptance among communities, and (c) favorable perceptions/competitive advantage in securing contracts through increased engagement in humanitarian health response activities. Formal intersectoral dialogue is needed to clarify opportunities for coordination at the prehospital level through mechanisms including Ministries of Health, locoregional health systems, and professional societies, and the WHO Emergency Medical Teams Initiative, all of which will be subject to contextual variability.^23^

Current practices for in-country health facility capacity assessment and mapping were felt to lack standardization and coordination. Participants described ad hoc relationships established with local surgeons/anesthetists for referral coordination at the health facility level. Participants also highlighted the redundancy of current practices in which each mine action operator performs their own organizational site visits and capacity evaluations. There was limited-to-no coordinated dialogue or engagement with ministries of health on this topic. Opportunities exist to streamline this process. A standardized process for facility mapping could be established with a mechanism for information-sharing between HMA stakeholders, thereby reducing redundancy and resource utilization expended during capacity assessments. These data should be shared and ultimately collected in collaboration with national Ministries of Health. Coordination could be facilitated by the Mine Action Area of Responsibility at the country level in collaboration with national mine action authorities if adequate capacity were devoted to victim assistance issues. Closer health stakeholder engagement would be mutually beneficial through leveraging of existing information in the WHO Health Resources and Services Monitoring Availability System, other WHO standardized assessment tools, and health cluster data, as well as improved integration with local health authorities such as ministries of health. IMAS 13.10^b^ mandates mine action operators to collect data on relevant existing services in operations to help develop a directory of services compiled by the relevant government entity. Once synthesized, this facility mapping could be fed back to communities and LFR trainees to ensure clear notification pathways for EO/EW casualties.

This study had several limitations. First, study participants were recruited utilizing convenience and snowball sampling strategies, which may limit representativeness of the perspectives incorporated. This recruitment strategy, sample size, and sector-specific nature of the study’s objectives may limit generalizability and lead to premature saturation of themes. However, the sample represents a broad set of stakeholders with extensive experience in policy, health, and HMA including victim assistance. Second, voluntary response bias may be present, and those with less positive views of engagement between HMA and civilian casualty care may be less likely to be represented. This risk is felt to be low given that participants expressed both positive as well as potentially negative aspects or challenges encountered in coordination between HMA and emergency care for EO/EW victims. Third, study findings will require adaptation to specific local contexts and conflict dynamics. Our research group is engaged in ongoing work to address this gap, specifically a pilot of joint LFR trainings with EORE among IED-affected communities in Burkina Faso.^12^ Finally, while long-term physical and psychosocial rehabilitation for EO/EW victims is essential, the focus of this article is on strengthening emergency care to ensure that patients survive to reach the rehabilitative phase of care. Therefore, considerations surrounded strengthened long-term rehabilitation were not included in this study. Despite these limitations, our study provides current attitudes and perceptions around opportunities to enhance coordination between HMA and the emergency health response to EO/EW casualties that may be used to improve outcomes for civilians with explosive injuries in conflict and post-conflict settings.

## Conclusion

Numerous opportunities exist to leverage medical capability present within humanitarian mine action to support emergency care strengthening in settings affected by EO/EW to reduce preventable death and disability among casualties. Key priorities include integration of layperson first responder trainings with explosive ordnance risk education activities and strategic utilization of mine action medics to support trauma care training for prehospital personnel. In addition, the benefits of service mapping by the mine action sector and the communication of locations where emergency medical should be reinforced, as per IMAS 13.10, can be leveraged to achieve improved coordination between HMA and health actors for mapping of in-country health facility capabilities with associated prehospital notification pathways and structured casualty referral. Increased support for the emergency care elements of victim assistance is required to facilitate these objectives. Clear mechanisms for integration of both layperson first responders and HMA personnel into local health care governance frameworks is required. To effectively achieve these goals, close collaboration and long-term coordination strategies with Ministries of Health and local health care personnel must be established.

## Supplementary Material

1

## Figures and Tables

**Table 1.** Interview participant characteristics

	*N*	%
Demographics		
Male	12	63
Female	7	37
Organizational affiliation		
Humanitarian mine action NGO	11	58
Medical or other humanitarian NGO	3	16
Academia	3	16
Government agency	2	10
Policy and advocacy	1	5

NGO – Nongovernmental organization

**Table 2.** Selected quotes by theme

Phase of care	Theme	Quote
Layperson first response	Prolonged prehospital times; lack of organized, effective prehospital systems	“The road network was really bad, and it was very hard to get from place to place in a reasonable time…It was only aimed to be a measure mitigation measure, obviously, not the medical care. But this idea of getting more quickly to people, right, in order to do some basic things that might help extend their life or prevent their injuries from being worse.”
		“He spent 27 hours on route to get to a hospital after his injury, and that’s not an uncommon thing.”
	Bystanders frequently first on-scene	“So you can train first aid responders, they can come in, they can bandage someone up, someone’s got a motorbike or something, you can throw them on the back of it and get them out. And as long as they can take the 5-hour ride on a motorbike, then they get to the health center.”
		“And we checked basically, how do people get to hospital? …This usually is a normal taxi driver.”
	Importance of responder safety	“You need to build in a very good risk education component for the first responders, so that they don’t run into the minefield.”
		“…our primary target audience are the ones that are going out and doing this because they will not have a health care provider who’s immediately available. Depending on where they are, it could take a very long time for the ambulance or the systems to get there…So in terms of liability in those cases, they’re already there. They’re already the ones that are in the area of the contamination and they’re risking their lives so we’re not asking them to do something extra. We’re just trying to help them mitigate the risk in that situation that they’re already in…they’re going in there anyway. So what we do is simply just providing them with some tools to try and reduce the risk.”
	Sustainability concerns and equipment	“Taking into account what are the likelihood of the resources they will have available down the track for using the skills, so we do provide some equipment when they get this training. But being mindful that in most of these contexts, we do not have a system for ensuring that this is replenished when they’ve used it.”
Prehospital	Underutilization of demining medics	It does feel like a bit of a waste that quite well-trained medical people sat there for 8 hours a day. Clearly there’s a requirement. You can’t distract them with intensive training, but basic sort of ABCs of first aid and things like this… They could quite easily cover that without losing focus on that day-to-day task.”
		“…there’s scope for that, of saying, right, if you’re there in a place for 2 months, for example, maybe 1 afternoon a week, the paramedics could change sections. And you could rotate around and do the moulages and the training that we do for the for the paramedics, and teaching for some local groups who would be useful to have an uptake of that training. And I think it would be advantageous for [organization] in terms of trying to gain contracts to show that you are moving outside of just demining but moving into more humanitarian health care, in the context of conflict and where there’s explosive ordnance…So that for example, we could then carry on working on the minefield, and then have one afternoon a week, for example, people training local staff in a health clinic about trauma care.”
	Ad hoc nature of current engagement with civilian casualties	“Particularly in remote areas, our medics do get involved with the local community a lot anyway. But normally outside of that 7-hour mining window. Yeah, so that can be on the weekends or, you know, during stand down periods, where our medics, or it might be our American medical advisor who gets involved rather than the medics themselves….that’s on a very ad hoc basis. But you could do that a bit more systematically.”
Facility-Based	Redundancy/inefficiency in health facility mapping and referral pathways	“Of course, we need to always prepare for the worst, and we need to map where is the functioning medical service and at which levels. We need to understand the levels of medical care what they can offer, what they cannot, what is the fastest way of emergency evacuation.”
		“We need to know if there’s a local clinic… If not, well, then we need to plan our evacuation routes another way. And then we start to understand the victims’ situation.”
		“No, we do [facility mapping] internally. So we would go and visit the hospitals individually. So for example, I’m in [x] area. I’ll go to visit the hospital and often get a relationship with the top 1 or 2 there. Now, occasionally this can be a bit of a quid pro quo…”
	Gaps in surgical competency and resource availability for EO/EW casualties	“If they’re treating a trauma, we can never guarantee that the hospital will have supplies that are needed for a particular point in time.”
		“We see a lot of people going without the surgeries they need, either there’s not the surgery, they can’t afford it, and so you’re really challenged with a lot of those kinds of things, too, and especially as you get into any kind of specialization, lots of general surgeons out there, but general surgeons don’t always know the best way to make a stump, you know. And that can be really challenging for somebody who wants to have a prosthetic device.”
	Opportunity for bidirectional refresher trainings	“What’s useful is having our chief medic or assistant medic, or even some of the good paramedics, cycle through the emergency department or the wards of that hospital. So they can get a bit of an idea of how the hospital system can run. So I will normally write that into the program for the chief medic, that 1 morning or 1 day a week, then 1 day a month, you’ll go to the hospital and just pick up and work with their doctors, nurses in whichever Ward and see how that goes. That’s quite useful for nurses that have been out of the system for a long time. The pool of medics and chief medics, as you can probably imagine, doesn’t always come from practicing nurses or practicing doctors…”
Data Collection and Quality Improvement	Inadequate operationalization of data collected	“There was a real concern early on with doing surveys of landmine survivors that you survey, and you find out what their needs are, and they don’t get any benefit. They don’t see any more services. They don’t see it making it easier for them to get to the clinics.”
		“Some of the major challenges were raising awareness of why data like this is important, how it shapes what the response is…We have a lot of discussion about what should be on the survey form that the data collectors are using, what is important information, what is less important information, what should be that common core of information that we should require all programs to collect?”

**Table 3.** Recommendations by phase of care

Phase of care	Recommendation	HMA implementation strategy	Interface with WHO and other health toolkits	Notes
Layperson first response	Implement and scale LFR trainings	Integrate LFR trainings with community-based EORE activities	Utilize conflict-adapted version of WHO CFAR curriculum	Ensure robust scene safety training and adequate provisions to prevent layperson first responders from becoming secondary victims
Prehospital	Prehospital training	Utilize medics either at periphery of worksite or after hours/dedicated shifts to support training of prehospital personnel	WHO-ICRC Basic Emergency Care (BEC) Course Module on Conflict-Related Injuries; WHO Basic Ambulance Provider Course; ICRC Blast Trauma Care (BTC) Course	Ensure appropriate agreements in place with local health stakeholders/professional societies/Ministry of Health. Ensure agreements/legal frameworks present for HMA personnel to be able to utilize their clinical expertise in-country.
	Facility mapping and notification systems	Coordination with health stakeholders for shared facility mapping processes with associated notification pathways to be distributed through community liaisons	WHO HeRAMS and health cluster data; WHO Hospital Emergency, Critical and Operative Assessment Tool	Geospatial capabilities and data present within Information Management for Mine Action (IMSMA) can be leveraged
Facility-based	Trauma team organization and activation/resuscitation rehearsals	Bidirectional refresher training in health facilities for mine action medics with coordinated support to facility-based trauma care providers	WHO Trauma Care Checklist; WHO BEC; ICRC BTC; WHO Emergency Care Toolkit (24)	HMA paramedics can catalyze organization of trauma teams with designated roles and rehearsals of trauma resuscitations to improve care quality
	Support for operative training focused on blast specific injury patterns (e.g., amputation techniques, burns)	Programmatic advocacy and opportunity for mine action medical personnel exposure	WHO Operative Care at the First Level Hospital; IGOT SMART Course; Burn care trainings for low-resource environments (25)	Limited direct clinical engagement from HMA given surgical skills required, but may be appropriate for paramedics
Data Collection and Quality Improvement	Improved operationalization of casualty data	Clarify pathways for secure data sharing with health stakeholders to improve planning of trauma care for EO/EW casualties	WHO EMT minimum dataset; WHO Clinical Registry	Ensure data privacy and security measures upheld through establishment of data sharing agreements

BTC – Blast Trauma Care; CFAR – community first-aid responder training; EMT – Emergency medical teams; EO – explosive ordnance; EORE – explosive ordnance risk education; EW – explosive weapons; HeRAMS – Health Resources and Services Availability Monitoring System; HMA – Humanitarian Mine Action; ICRC – International Committee of the Red Cross; IGOT – Institute for Global Orthopaedics and Traumatology; LFR – layperson first responder; SMART – Surgical Management and Reconstructive Training; WHO – World Health Organization.

## Footnotes

a Section 5.2.5

b Sections 5.1.2, 5.2, 5.2.3
