# Supplementary material for: A Qualitative Analysis of Opportunities to Strengthen Coordination Between Humanitarian Mine Action and Emergency Care for Civilian Casualties of Explosive Injury
Source: Disaster Med Public Health Prep. Author manuscript; Available in PMC 2025 Aug 20. (PMC11928725; doi:10.1017/dmp.2025.30)
Supplement: 1 [file NIHMS2050312-supplement-1.docx]

**Supplemental information**

Supplement 1. Semi-structured interview guide

**Supplement 1. Semi-structured interview guide**

Intro

- Introductions
- Briefly present project rationale and background

Questions

- In your experience, how do mine action groups currently interface with emergency health response for victims of blast injury?
  - What degree of awareness/overlap is there between demining operations and civilian casualties of blast injury?
- What differences have you observed in various crisis settings?
  - Which actors/individuals/local partners are most important for providing an understanding of context-specific dynamics?
- What types of projects do you feel mine action groups and country partners would be most interested in developing and maintaining related to post-blast care?
- What types of data are collected by your organization, and how are these used?
- What previous programs or interventions have you seen related to medical care of victims of blast injury?
  - Can you please share any successful examples, and what factors led to success?
  - Can you please share any unsuccessful examples, and what factors led to failure?
- What do you feel are the most significant barriers to engaging mine action services in the provision of post-blast medical care?
  - Have issues of liability been raised as a concern in the past, and if so, could you please expand on this?
  - What are your thoughts around liability related to training lay first-responders, who may then be going into dangerous areas to reach casualties?
- What opportunities do you think exist to engage mine action services in post-blast medical care, and to improve the coordination between mine action services and emergency health response?
  - How might it be possible to synergize efforts to improve the care of civilian casualties with priorities of mine action groups, so that such efforts are not viewed as an additional burden on operations?
- What considerations around implementation readiness are critical for such an intervention to be feasible, context-appropriate way?
- What other ideas or experiences you would you like to share?

Conclusion

- Can you please provide the names of any additional individuals/stakeholders you recommend we engage in this process? Would you be willing to facilitate any of these introductions?
- Discuss opportunities for follow-up conversations and further engagement
